# HOW THE PATIENT-CENTERED MEDICAL HOME MODEL IMPACTS OLDER ADULTS’ ACCESS TO HEALTH CARE IN RURAL GEORGIA

**DOI:** 10.1093/geroni/igad104.2470

**Published:** 2023-12-21

**Authors:** Elisa Childs

**Affiliations:** University of Iowa, Iowa City, Iowa, United States

## Abstract

Rural Americans face several healthcare access barriers, including limited providers and driving further distances to receive care. Georgia healthcare access is especially problematic given its high rurality, hospital closure rate, number of medically underserved areas, and older adult population. The government promotes the Patient-Centered Medical Home (PCMH) model to improve healthcare access. This study supports a better understanding of older Georgian’s access to care by exploring PCMH’s ability to improve healthcare access. Older adults were surveyed using a modified version of the Healthcare Quality Survey (N=746). Analyses included univariate, bivariate (t-test, ANOVA, chi-square, and a crosstabulation), and multivariate (multiple linear regression). Healthcare access was statistically significantly different depending on care source type, with significant associations between PCMH users and income, age, urban/rural status, and health status. Access was also statistically significantly different based on annual household income, educational attainment, age, and health status. Further, a significant relationship was found between access and age, income, and health status. PCMH users were primarily Caucasian and most often had an advanced degree and income of $60,000 or more. PCMH users most often reported living in Three Rivers, Atlanta Region, Northeast Georgia, and the Georgia Mountains. Finally, urban and rural residents with a regular care source reported “always” receiving needed care more often than PCMH users and those with no care source. The National Committee for Quality Assurance and other accreditation agencies should target low-income rural areas with high older adult populations when implementing the PCMH model to increase older adults’ healthcare access.

